# The Dental Pulps

**Published:** 1878-12

**Authors:** Wm. A. Pease

**Affiliations:** Dayton, Ohio.


### THE DENTAL PULP.

BY WM. A. PEASE, DAYTON, OHIO.

The consequences of the exposure of the pulp of a tooth are serious to the patient, perplexing and unsatisfactory to the dentist. That they should have remained so to this date is evidence of the difficulties and complications that surround them, and the ignorance of the best means to overcome them.

Thus far they have been disappointing; nothing has yet been found that seems effectual only in exceptional cases, unless the exposure is recent and slight. Several materials have been used which the experimenters believed had some general efficacy; their opinions probably being colored by hope and the novelty to them of the materials used; as the experiments, carefully repeated by others, failed to produce valuable results. After the application of them the pulp has not always died immediately, or, having died by the suddenness or severity of the application, quietly or unobtrusively, the effects of it were smothered by some antiseptic properties of the materials used, until the pulp was dessicated, or the case finally passed beyond observation. Occasionally the pulp has survived the most heroic and exasperating treatment; and we have heard of it in our societies or read of it in our journals, and many dentists, overjoyed at the result, have rushed to treat every exposed pulp, without discrimination in the same manner, and without stopping to inquire if it were rational or even probable that the known properties of the materials were suitable for the case. Especially has this been the case if the dent st has obtained some notoriety for facility and frequency of debate in the societies on every subject, and his writings, clothed in words idiosyncratic of his temperament and merited training, have seemed oracular, to have some pith in them, and have been repeated with bewildering frequency in the journals.

There are some things that are self-evident and non discussable after the mastery of the elements. One of these is that the exposed pulp should never be irritated; that all our efforts to save its life should be soothing, quieting. Entering the tooth by a minute thread it expands to considerable dimensions before terminating in all, and in some teeth in an enlarged bulb, which requires an active circulation to nourish it. This makes it analagous to erectile tissue, in this, when subject to irritation, that it becomes suddenly engorged, but unlike it, it has no means of instantly relaxing when the excitement is past; and the consequences are congestion, blood, stasis and death. Let me enlarge a little, even at the expense of seeming to explain what is axiomatic. In the normal condition of the tooth the channel for the entrance and exit of the blood through the foramen is just equal to the healthy demands, and there is no useless tissue between the afferent and efferent vessels—the nerve and the walls—nothing to yield, to accommodate an increased volume of inflowing blood, which must impinge upon and diminish the capacity of the outflowing vessel; and consequently any irritation of the pulp at the point of exposure must be responded to by an increased volume of inflowing blood that can not rapidly escape; and hence the excruciatingly spasmodic action of the nerve to relieve itself from the pressure of the expanded blood vessels, and the frequency of its death under otherwise very slight irritation. We all know its extreme sensibility to heat and cold, sweets and sours, or to anything acrid. Encased in bony walls of but little conductivity, what must be its astonishment and protest at the sudden invasion of its premises by the rapidly cutting drill of the engine, an element of exposure much more considerable than that of the hand and which makes this discussion more important and imperative. It is the healthy, exposed pulps that is now under consideration. The pulp is exposed, a condition pregnant to the thoughtful dentist with serious considerations, perhaps of self-upbraidings. What will be done with it? Kill it and leave the patient to all the possible and probable consequences of it; the shortest way, or attempt to save it—almost a forlorn hope. If we attempt to save it, what are our means? Those taught by the books and those used by the most considerate operators—are they rational; such as we would apply to the eye or an infant's mouth and expect no irritation; because irritation of the pulp is death; it may not be immediate, but it is generally quick and sure. Let us look at Kreosote and Carbolic Acid, analogous, and probably used more than any other means. What should we expect from the use of either of them on a tissue such as has been described? They are escharotic on live tissue and antiseptic on dead. A singular remedy for so tender an organ as the pulp. Are they what we want? Shall we eat a hole into the pulp, hoping it will eventually cast off the eschar and get back to the point of beginning after the tooth has been filled. Where will it cast it to, or what will become of it? Will it lie an irritating substance between the filling and the pulp and pressing upon it? Will the pulp endure that, or will it be absorbed? If absorbed, the pulp has greater absorbing powers than any other parts of the system and there is no reason, situated as it is, why it should have any, unless Nature anticipated that there should be den'ists, and that they should apply Kreosote to it; as the same matter, in the same form and condition, antisepticised, inserted under the mucous membrane or the skin, would not be absorbed; but it would come to the surface by ulceration. Besides, we can not see why Kreosote or Carbolic Acid should first have been used by a dentist, unless empyrically and in desperation. They were probably brought from a nursery, where they are used indiscriminately and unconsciously for two purposes. As an escharotic they relieve pulpitis by lessening tension, and as antiseptics they relieve incipient periostitis by stopping the formation of gas. But to use either of them as an ingredient of a cap, leaving it there to simmer, vaporize and eat into the pulp is simply to drive it into fits without any possibility of curing them. If this reasoning be correct then any irritating material is objectionable in proportion to its irritating quality. This will include all of the tinctures, even though they carry sedatives; as the action of alcohol is instantaneous and irritating to a healthy surface. Having examined the usual remedies and found them objectionable in principle and rarely efficacious, we find we have limited our means very much, without having offered anything as a substitute that experience has sanctioned. Perhaps the most satisfactory material of recent trial is white lead, white lead and chalk or Creta Prep alone; each and all of them carrying a sedative in watery solution, and applied directly to the point of exposure, to be capped by a bit of sheet lead. If time will admit, a sedative, as a little of morphine applied to the place an hour before is desirable; then cap as above; cover with os artificial and there should be little or no pain; if the materials are not too flowing and the cap is large enough to shield the sensitive parts. Here there is no pressure and nothing acrid; the little chalk that escapes from under the lead cap helps to neutralize the acid of the os when the bone is thin; when it is thick there is no need for it; but where practical the lead cap should cover all of the bone nearest to the pulp. All of these materials are soothing except the os, and it should be kept out of the way of harm; and when the nerve awakes from its slight narcotism it should be bright and buoyant, ready for everything it has to do. Sometimes I have omitted the os and plugged over the lead cap with amalgam; and at others have cut a thin piece of spunk to the proper shape, struck it with a hammer to condense it, laid it over the lead and plugged with gold or amalgam. Soon after I commenced using spunk I was filling the second lower molar with gold when it commenced aching severely. The filling was removed but no exposure could be found. It was evident from trials the filling could not be borne, and the temperament and age were not promising for destroying the pulp; here was a dilemma. I saw the spunk just used for drying; cut a piece as described above, covered the bottom of the cavity with it, touched it with a particle of Kreosote to correct effusion, if any, and the filling was a success. It gave a very comfortable feeling to the patient; and in favorable cases of slight exposure is a success; as it prevents those distressing spasms from sudden thermal changes. I have been in the habit of using this for more than fifteen years with more satisfaction than any other material; but the exposure must be slight, and it must be used with discretion.

The death of the pulp after filling the tooth, when no exposure exists, or where it is very slight, is too frequent; but where exposure exists it must be expected in many cases, unless the greatest discrimination is used in regard to the teeth and the patient. Pulps die quietly, and without aching, in teeth where they have been and have not been exposed; and their taking off is probably greater just before exposure, in abraded teeth, and where decay has been slow. It must be expected, therefore, that the sudden changes incident to the preparation of the cavity and the insertion of a metallic filling must materially increase the mortality. These deaths have given endless trouble to dentists, which has served to fix the fact in the mind to such a degree, that many of them believe there is a tendency in the pulp to die; that at some indefinite period they cease to be useful to the tooth; that they then gradually withdraw, filling their chambers as they retire with bone.

They assume the effect to be the cause, the necessity, the reparatory effort to be volition, obedience to a natural and predetermined law. The effort of Nature for self-preservation, to be a voluntary abdication of function—a negation of the harmony and equilibrium of the system; the introduction of a disturbing element, to plague and embarrass the assimilating and emunctory organs, at a time of life when they most need all the auxiliaries that the system can provide. They can not see in it a physiological action; a baricade against a physiological danger, a supreme effort at self-preservation, and a wonderful choice of means, never used unless as precautionary to guard against aggression. It is not made for self alone, but to guard the house it inhabits from other dangers and other foes. The loss of the pulp begets periosteal complications, that may result in necrosis, by which I mean not exfoliation, or acrid and gradual solution, but denudation and an inability to receive nourishment for a longer or shorter space, beginning at the apex of the root. Complete denudation is absolute death, when the tooth must be cast off as a foreign body; and partial denudation necessitates the intervention of a cyst to protect the soft parts by its fluid contents, as a cushion from puncture or laceration from the point of the bare root. Wherever aberration or loss of function takes place in any organ it does not stop there, but the sequences of it are almost unending. The death of the pulp is always accidental or circumstantial, not due to heredity or an acquired or natural tendency. The teeth that are called permanent, were not designed to be shed at any indefinite period during life, or to have their usefulness injured by periosteal complications. Whenever these occur they are adventitious, not contemplated by nature, and are due to accidents and diseases of life. Most of the gum and alveolar diseases are due to carelessness or negligence, the administration of improper medicines, or to a hereditary cachexia, that was acquired by more or less remote ancestors. There is nothing in the teeth, structural or otherwise, or in the surrounding parts, that implies that the tooth tends to obliteration; that it grows feebler and feebler, in any greater ratio than the other parts of the system; that at any time its service can be dispensed with without material detriment; the institution of a chain of sequences, whose detrimental consequences can hardly be estimated. What is the natural type of the teeth? Such as we see now, or occasionally get glimpses of, large, solid, strong, beautiful as minted gold, and almost as enduring, or better yet, better than dentist ever saw; so perfect that the fruits, grains, meats, unaided by cookery, should be preferred by them for sustenance and a vigorous life. Could the first man have lived plagued with the toothache, without mastication and bolting his food, to the age that he did, if the pulp tended to obliteration then, as it is said to now? Would the average teeth of the people of our cities serve for that purpose? Have they improved in structure and health under our modern mode of life and cookery, which deteriorates them by disuse?

Whilst nature did make provision for the accidental loss of the teeth, by providing for the absorption of the socket, which must otherwise have remained open and foul, there is no evidence that she contemplated it on a large scale or otherwise than an accident. She protected the pulp as she protected the important nerves and arteries, in an inaccessible place, deep from danger. The known longevity of the pulp, which is not exceptional, the tenacity with which it clings to life, when its home is scarcely habitable, and the disastrous consequences following its loss, for which human ingenuity has not yet found a remedy, all lead to the inevitable conclusion that nature intended it to stay in the tooth through life. This conclusion is fortified by the complex plan of the teeth, as to purpose and function, arranged with reference to the wants of a being of the highest organization, and the most diverse needs, and for the preparation of food, embracing the greatest variety of materials for incision, tearing, grinding, an apparatus more complete than any other animal has, and comprising all of the peculiarities and excellences of each. Its completeness shows its need, not for youth, not for middle age, but for all life, for the complex, sensitive and easily disturbed organs standing back of it, depending on its complete discharge of function for the discharge of theirs. Caries may invade its habitation and it succumbs, not without a terrible protest; abrasion may slowly invade it and it cautiously erects baricades against it, and does all within its limited means for protection, but this comes in few instances and late in life, and it is either without the normal plan of nature or an accident. Incision to be perfect demands that the incisors should pass each other and not meet in direct apposition, to be constantly dulled and worn out of edge. This is the usual arrangement of the human incisors and canines; the edges lap by each other, and if there is any wear it is to sharpen them and not to blunt. The exceptions are few and exceptional, and rarely congenital. The exceptions generally arise from the loss of the molars and bicuspids, which gives a longer sweep to the lower jaw, and brings the incisors into constant use and apposition, and the ultimate abrasion to the pulp. This happens late in life, while the devitalization from caries and other causes is generally early.

Forty years ago my medical reading and oral teaching was that the teeth of very old persons had no pulps in them; that at some indefinite time in their lives the pulp disappeared, become obliterated, filled up and the tooth solid, and as I then thought, painless; for at that time, if I recollect rightly, we were not instructed about periostitis. Having an aching tooth then, I recollect that I thought that if age had some inconveniences it also had some compensations. But this theory was of short duration; for among the first teeth I extracted was two for husband and wife, both over ninety years old, and both had healthy pulps in them. They were opened with eagerness to see how a pulpless tooth, with the cavity obliterated would look; and great was my disappointment to find not only a healthy tooth but a normal chamber for it to live in. Since then I have broken several pairs of forceps in splitting teeth for a special study of their pulps.

More than forty years ago I had a bicuspid filled in both its front and posterior faces; one of the fillings was subsequently removed and about twenty years ago the crown broke away leaving a sharp edge on the labial and lingual faces. I went to a dentist who has recently added to his reputation by his writings on the diseases of the gums, to have it filed smooth. He advised extraction, said it was too short to be of any use, it would probably be troublesome, etc., but he consented, saying, "You know what you are about." To-day I have examined that tooth, and bit a piece out of my finger nail with it. It is slightly crescent shaped, the surface bright and polished except a slight stain on one edge frequently seen on molars that have lost their enamel; and it is now on edge from having eaten sour apples. It has never ached, and it has as lively a pulp as need be, though the house it lives in is dilapidated and circumscribed, I am now building up the crowns of the six front teeth, for a gentleman about seventy years of age, that were so abraded before he consulted me, that the pulps were dying. They were removed having just began to generate gas, but they were yet plump and whole.

These examples might be multiplied indefinitely; they are of frequent occurrence and they are not exceptional. I have been thus particular to show that the pulps may die like human beings from little or great provocation at any time from youth to old age; but that they have no predisposition that way; that if they do not hanker after longevity they are at least content to live as long as the house that contains them is protecting and habitable. The theory that seems to be getting introduced how I think is pernicious, viz: that the pulp tends to obliteration, to die on the slightest provocation. The consequences of this is a want of study, a slovenly and harsh way of treating it, because whatever may be done, its tendences being to death, it will be ineffectual.

It is this doctrine of fatality I wish to correct in order to give life to investigation. It seems to be evolved more from the inner consciousness, than accurate and prolonged observation. Like the vaticinations of the Roman sooth sayers, from an inspection of the entrails of birds, this inspection of all the folds and duplications of the primordial membranes may give an equivocal omen, that may be interpreted in two ways, or, at least, give rise to mortifying exceptions. I hope it has been misinterpreted. I can not believe that nature intended that the pulp should die, while the tooth is habitable, or, that the permanent teeth have a tendency to be shed during the life of man. And I hope some of the means now used may be found effectual in saving the life of the pulp or that others may be found by further study.

In this paper I have noticed some of the means used for saving the life of the pulp, and some of these I deemed objectionable. In another I shall notice some of the means used for treating a tooth after the pulp is dead; after which, the way having been prepared for it, I hope to notice the measures and methods of filling the teeth and what may be expected from them.

